# Prioritization of *PLEC* and *GRINA* as Osteoarthritis Risk Genes Through the Identification and Characterization of Novel Methylation Quantitative Trait Loci

**DOI:** 10.1002/art.40849

**Published:** 2019-06-27

**Authors:** Sarah J. Rice, Maria Tselepi, Antony K. Sorial, Guillaume Aubourg, Colin Shepherd, David Almarza, Andrew J. Skelton, Ioanna Pangou, David Deehan, Louise N. Reynard, John Loughlin

**Affiliations:** ^1^ International Centre for Life Newcastle University Newcastle upon Tyne UK; ^2^ Freeman Hospital Newcastle upon Tyne UK

## Abstract

**Objective:**

To identify methylation quantitative trait loci (mQTLs) correlating with osteoarthritis (OA) risk alleles and to undertake mechanistic characterization as a means of target gene prioritization.

**Methods:**

We used genome‐wide genotyping and cartilage DNA methylation array data in a discovery screen of novel OA risk loci. This was followed by methylation, gene expression analysis, and genotyping studies in additional cartilage samples, accompanied by in silico analyses.

**Results:**

We identified 4 novel OA mQTLs. The most significant mQTL contained 9 CpG sites where methylation correlated with OA risk genotype, with 5 of the CpG sites having *P* values <1 × 10^−10^. The 9 CpG sites reside in an interval of only 7.7 kb within the *PLEC* gene and form 2 distinct clusters. We were able to prioritize *PLEC* and the adjacent gene *GRINA* as independent targets of the OA risk. We identified *PLEC* and *GRINA* expression QTLs operating in cartilage, as well as methylation‐expression QTLs operating on the 2 genes. *GRINA* and *PLEC* also demonstrated differential expression between OA hip and non‐OA hip cartilage.

**Conclusion:**

*PLEC* encodes plectin, a cytoskeletal protein that maintains tissue integrity by regulating intracellular signaling in response to mechanical stimuli. *GRINA* encodes the ionotropic glutamate receptor TMBIM3 (transmembrane BAX inhibitor 1 motif–containing protein family member 3), which regulates cell survival. Based on our results, we hypothesize that in a joint predisposed to OA, expression of these genes alters in order to combat aberrant biomechanics, and that this is epigenetically regulated. However, carriage of the OA risk–conferring allele at this locus hinders this response and contributes to disease development.

## Introduction

Osteoarthritis (OA) is a common, age‐related disease of synovial joints characterized by the thinning of articular cartilage. This thinning ultimately results in focal loss of cartilage and a full‐thickness lesion exposing underlying bone 1, 2. Cartilage loss prevents the joint from withstanding normal mechanical load and leads to severely impaired joint function. The clinical effect is a painful chronic morbidity and, in those with hip and knee OA, an increased risk of premature death due to secondary cardiovascular events resulting from reduced physical activity 3, 4. Regarding treatment options for hip and knee OA, there are no disease‐modifying OA drugs, and arthroplasty of hips and knees is a common, but not a risk‐free, procedure. As the population ages, the prevalence of OA increases. Novel treatments are therefore urgently required.

The causes of OA are complex and, with a heritability of >40%, genetic susceptibility is a main driver 5. Genome‐wide association studies have revealed that the OA genetic component is polygenic, with the association signals typically having modest odds ratios of <1.5 6.

Of those OA risk–conferring loci that have so far been functionally investigated, the vast majority mediate their effect by modulating the expression of a nearby gene. Clear examples include the single‐nucleotide polymorphisms (SNPs) rs143383, rs225014, and rs3204689, which correlate with differential expression of the OA and non‐OA risk alleles in cartilage of the genes *GDF5*,* DIO2,* and *ALDH1A2*, respectively (for review, see ref. 6).

DNA methylation at CpG dinucleotides is an epigenetic process used by the cell to regulate gene expression, and it can amplify or attenuate the impact of an expression quantitative trait locus (QTL) 7, 8, 9. Our group has previously shown this in the context of *GDF5*, rs143383, and CpG sites located close to the SNP 10. Identifying *cis*‐acting CpG sites, where methylation correlates with an association signal, can therefore offer insight into the mechanism by which the genetic risk at that signal operates. Such CpG sites are known as methylation QTLs (mQTLs). Furthermore, if mQTL CpG sites cluster within or close to a particular gene, their mapping prioritizes that gene for further investigation as a plausible target for the association signal 11.

We have shown the potential utility of this approach in our previous analysis of 16 OA loci, in which we correlated cartilage DNA methylation with OA association signals using genome‐wide DNA methylation and genotyping array data 12. In this current study, we extended our investigation to 18 novel OA association signals that have been identified in the last 3 years. We found evidence of mQTLs at 4 of these loci, with the most statistically significant effect being on chromosome 8q24.3 at a signal harboring a number of genes. We subjected this signal to in silico and in vitro analyses, which highlighted the plectin gene *PLEC* and the TMBIM3 (transmembrane BAX inhibitor 1 motif–containing protein family member 3) gene *GRINA* as the targets of the association. Plectin is a multifunctional cytoskeletal protein that is particularly abundant in tissues subjected to mechanical load and stress, while TMBIM3 is involved in the control of cell death by endoplasmic reticulum (ER) stress. Our study highlights the high frequency of cartilage mQTLs operating on OA risk loci and the capacity for mQTL analysis to prioritize a gene from within a gene‐rich region as a target of the association signal. *PLEC*,* GRINA,* and their encoded proteins now merit much more detailed analyses in the context of OA genetic risk and cartilage biology.

## Materials and methods

#### Cartilage methylation and genotyping array data

We used cartilage CpG methylation and genotype data that we had previously generated using an Illumina Infinium HumanMethylation450 array and an Illumina HumanOmniExpress array, respectively 12. We had both methylation and genotype data available for a total of 87 patients who had undergone knee or hip joint arthroplasty (57 knee OA patients, 14 hip OA patients, and 16 patients who had undergone hip replacement due to a femoral neck fracture (Supplementary Table 1, available on the *Arthritis & Rheumatology* web site at http://onlinelibrary.wiley.com/doi/10.1002/art.40849/abstract). The cartilage samples from the patients with femoral neck fracture lacked OA lesions.

#### OA loci investigation and mQTL analysis

We investigated 18 novel OA risk loci that had been reported as being associated with the disease at a significance level close to or surpassing the genome‐wide threshold of *P* < 5 × 10^−8^ 13, 14, 15, 16, 17, 18, 19, 20 (Table 1). If the OA‐associated SNP was directly genotyped on an Illumina HumanOmniExpress array, we utilized those SNP data. If the association SNP was not on the array, we identified and, where possible, used a proxy SNP that was in perfect or high linkage disequilibrium (pairwise r^2^ > 0.7) with the association SNP. Proxy SNPs were derived from a candidate list using LDlink's LDproxy tool (https://analysistools.nci.nih.gov/LDlink/) 21 and European population data. Where multiple proxy SNPs were identified, we chose the one with the highest r^2^ relative to the association SNP.

**Table 1:** List of the 18 osteoarthritis association signals in the study^a^

Locus	Association SNP	Allele, major/minor	MAF	Chr.	Nearest protein‐ coding gene	Proxy SNP (r^2^ relative to association SNP)^b^	Stratum^c^	Ref.	CpG probes from the methylation array, no.^d^
1	rs2820436	G/T	0.34	1	*ZC3H11B*	rs2605096 (1.0)	Any joint site	13	153
2	rs2862851	C/T	0.47	2	*TGFA*	rs1807968 (0.92)	Hip	14	526
3	rs6766414	T/G	0.25	3	*STT3B*	–	Hip	15	186
4	rs2236995	A/C	0.49	4	*SLBP*	No proxy at r^2^ > 0.7	Hip	14	–
5	rs11335718	C/‐ (indel)	0.08	4	*ANXA3*	No proxy at r^2^ > 0.7	Any joint site	13	–
6	rs4867568	T/C	0.48	5	*LSP1P3*	–	Knee	16	18
7	rs10471753	C/G	0.38	5	*PIK3R1*	rs6893396 (1.0)	Hip	14	201
8	rs3850251	T/A	0.29	6	*ENPP3*	rs4383836 (0.81)	Hand	17	206
9	rs833058	C/T	0.38	6	*VEGF*	No proxy at r^2^ > 0.7	Hip	18	–
10	rs788748	A/G	0.49	7	*IGFBP3*	–	Hip	19	229
11	rs11780978	G/A	0.42	8	*PLEC*	–	Hip	13	1,759
12	rs10116772	C/A	0.45	9	*GLIS3*	–	Knee and hip	20	108
13	rs496547	T/A	0.35	11	*TREH*	rs598373 (0.78)	Hip	14	784
14	rs4764133	C/T	0.35	12	*MGP*	rs12316046 (0.98)	Hand	17	259
15	rs754106	C/T	0.50	13	*LRCH1*	rs9534442 (0.85)	Hip	15	224
16	rs864839	A/C	0.33	16	*JPH3*	No proxy at r^2^ > 0.7	Any joint site	13	–
17	rs2521349	G/A	0.40	17	*MAP2K6*	rs2521348 (0.98)	Hip	13	138
18	rs6516886	T/A	0.29	21	*RWDD2B*	rs2832155 (1.0)	Knee and hip	13	104

a MAF = minor allele frequency; Chr. = chromosome.

b For single‐nucleotide polymorphisms (SNPs) not present on the HumanOmniExpress array, a proxy SNP that had the highest linkage disequilibrium with the association SNP and that was on the array was used to infer genotypes. The threshold was set at r^2^ > 0.7. Thirteen of the association SNPs required a proxy and, at the threshold, a proxy was available for 9 of the 13 SNPs (rs2820436, rs2862851, rs10471753, rs3850251, rs496547, rs4764133, rs754106, rs2521349, and rs6516886), but a proxy was not available for 4 of the 13 SNPs (rs2236995, rs11335718, rs833058, and rs864839).

c Stratum highlights the joint with which the signal shows association from the original genetic study.

d This value shows the number of CpG probes from the Illumina Infinium HumanMethylation450 array that are present within the 2‐Mb region surrounding the association SNP.

For each locus, we covered a 2‐Mb region encompassing 1 Mb upstream of and 1 Mb downstream of the association SNP. For each CpG site within the 2‐Mb region, linear regression was used to measure the relationship between methylation, in the form of M values, and genotype (0, 1, or 2 copies of the minor allele). For the purpose of this analysis, we defined any genotype–methylation correlations identified within this 2‐Mb region to be a *cis* mQTL. CpG sequences that were directly modified by SNPs were not included in the analysis. We used age, sex, and sample type (OA knee, OA hip, and femoral neck fracture) as covariates. All mQTL calculations were performed using Matrix eQTL software 22, which implements a false discovery rate (FDR) estimation based on the Benjamini‐Hochberg FDR procedure 23 and accounts for the number of tests performed. Methylation β values were used for the purpose of visualization.

#### In silico expression QTL analysis

The genotype‐tissue expression database GTEx (https://www.gtexportal.org/home/) was searched for expression QTLs (eQTLs) at rs11780978. The search was performed in July 2017.

#### RNA‐sequencing (RNA‐Seq) analysis

The expression of genes of interest was assessed using RNA‐Seq data that we had previously generated from the cartilage of 10 patients with hip OA and 6 patients with femoral neck fracture 24, 25. These patients are unrelated to the 87 patients used in the mQTL analysis. Details regarding the 16 patients can be found in Supplementary Table 1 (available at http://onlinelibrary.wiley.com/doi/10.1002/art.40849/abstract). Transcripts per million (TPM) values for each investigated gene were extracted using R (http://www.R-project.org/) and visualized using the ggplot2 library in R. Differential expression analysis between OA and femoral neck fracture cartilage samples was carried out with a Bioconductor software package DESeq2 26. Hypothesis testing was performed using a DESeq2 implementation of the Wald test.

#### New patients

Cartilage tissue samples were obtained from 104 new OA patients who had undergone joint replacement surgery at the Newcastle upon Tyne NHS Foundation Trust hospitals. The Newcastle and North Tyneside Research Ethics Committee granted ethical approval for the collection of tissue samples, with each donor providing verbal and written informed consent (REC reference number 14/NE/1212). Our patient ascertainment criteria and the protocols for extracting nucleic acid from cartilage have been described in detail previously 27, 28, 29. Further details regarding the 104 new patients can be found in Supplementary Table 1 (available at http://onlinelibrary.wiley.com/doi/10.1002/art.40849/abstract). Nucleic acid from these patients was used for genotyping, complementary DNA (cDNA) synthesis, allelic expression imbalance (AEI) analysis, and targeted CpG methylation analysis.

#### Genotyping of SNPs

SNPs were genotyped by pyrosequencing (rs11783799 and rs11136345), by restriction fragment length polymorphism (RFLP) analysis (rs11780978 and rs7819099), or by a real‐time SNP genotyping assay (rs9100) (catalog no. 4351379; ThermoFisher Scientific). The rs11136336 SNP is in perfect linkage disequilibrium (r^2^ = 1.0) with rs11783799; the rs11783799 assay was therefore used to genotype rs11136336. Pyrosequencing and RFLP polymerase chain reactions (PCRs) were performed using a G‐Storm GS4 Q4 Quad Block Thermal Cycler (Somerton Biotechnology) and the primers listed in Supplementary Table 2 (available on the *Arthritis & Rheumatology* web site at http://onlinelibrary.wiley.com/doi/10.1002/art.40849/abstract). Pyrosequencing assays were designed using PyroMark assay design software 2.0 (Qiagen), and the sequencing was performed using a PyroMark Q24 Advanced platform (Qiagen) with the recommended kit, following the instructions of the manufacturer. The RFLP‐digested fragments were separated by electrophoresis through a 3% agarose gel and visualized using ethidium bromide staining. The rs9100 real‐time genotyping assay was run on a QuantStudio 3 (Applied Biosystems), using TaqPath ProAmp MasterMix (ThermoFisher) following the instructions of the manufacturer.

#### Complementary DNA synthesis and AEI analysis

Complementary DNA was synthesized from 1 μg of cartilage total RNA using a SuperScript First‐Strand synthesis system (Invitrogen) and random hexamers following the standard protocol of the manufacturer after an initial 30‐minute treatment with 1 unit of Turbo DNase (Invitrogen) at 37°C.

AEI at transcript SNPs rs11783799 and rs11136345 was quantified by pyrosequencing, using the methodology described in the above genotyping section and the same primers. The sequences were generated automatically, and an output of allelic ratio was produced using PyroMark Advanced software. AEI at transcript SNP rs9100 was quantified using the above real‐time SNP genotyping assay. The use of such assays for AEI has been described previously 30. For each cartilage cDNA and DNA sample, PCRs were formed in triplicate. Samples were excluded from the analysis if the values between the PCR replicates differed by >5% for the pyrosequencing assays, or by >0.5 cycle thresholds (Ct) for the genotyping assay. The respective cDNA and DNA were analyzed concurrently, and allelic expression of cDNA was normalized to its corresponding DNA.

#### Targeted CpG methylation analysis

Methylation analysis of CpG sites cg19405177 and cg14598846 was performed by pyrosequencing using the methodology described above and the PCR and sequencing primers listed in Supplementary Table 3 (available on the *Arthritis & Rheumatology* web site at http://onlinelibrary.wiley.com/doi/10.1002/art.40849/abstract). Cartilage DNA was bisulfite converted using an EZ DNA methylation kit (Zymo Research). For each sample, PCRs were performed in duplicate and the mean calculated, with samples being excluded from the analysis if the methylation between the PCR replicates differed by >5%.

#### Chromatin interactions

The WashU Epigenome Browser 31 was searched to identify long‐range chromatin interactions extending from the CpG sites within the *PLEC* locus. All publicly available Hi‐C and long‐range chromatin interaction data sets were loaded for all cell types with available data within the genomic region chromosome 8:145,000,040–145,068,742. The region was searched visually to identify interactions stemming from either of the 2 clusters of CpG sites. The human breast cancer cell line MCF7 and the chronic myeloid leukemia cell line K562 interaction schema represent protein factor–mediated chromatin interaction data measured by RNA polymerase II chromatin interaction analysis using paired‐end tag sequencing (ChIA‐PET) data 32. These data were produced as part of the ENCODE project (https://www.encodeproject.org/). Data from the human lymphoblastoid cell line GM12878 were produced by RNA CCCTCF–binding factor ChIA‐PET 33.

## Results

#### Identification of OA mQTLs

Table 1 provides details of the 18 loci investigated. For 5 of the 18 loci, the SNP that had been used to identify the association signal had been genotyped on the HumanOmniExpress array. For each of the remaining 13 loci, we searched for a proxy SNP (r^2^ > 0.7). This was successful for 9 loci, but for the remaining 4 loci there was no proxy (loci 4, 5, 9, and 16). Therefore, these 4 loci were not studied further. In total, we analyzed 4,895 CpG sites across the 14 loci. We assessed correlations in all 87 samples combined for knee OA, hip OA, and femoral neck fracture. This analysis identified 4 SNPs that correlated with methylation (FDR *P* < 0.05): locus 7, SNP rs10471753 and 1 CpG site; locus 11, SNP rs11780978 and 9 CpG sites; locus 14, SNP rs4764133 and 1 CpG site; and locus 18, SNP rs6516886 and 6 CpG sites (Table 2 and Supplementary Table 4, available on the *Arthritis & Rheumatology* web site at http://onlinelibrary.wiley.com/doi/10.1002/art.40849/abstract).

**Table 2:** List of the significant genotype–methylation associations identified^a^

Locus [association SNP/proxy SNP if required] (Chr.), CpG site	CpG location (hg19)	*P*, uncorrected	*P*, FDR adjusted
7 [rs10471753/rs6893396] (5)	67586258	0.0001	0.03
cg25008444			
11 [rs11780978] (8)			
cg19405177	145001428	2.04 × 10^−20^	3.33 × 10^−17^
cg20784950	145002522	3.14 × 10^−07^	0.0001
cg01870834	145002835	2.05 × 10^−07^	0.0001
cg07427475	145008110	1.51 × 10^−18^	1.85 × 10^−15^
cg02331830	145008288	6.17 × 10^−11^	4.31 × 10^−8^
cg04255391	145008397	2.34 × 10^−17^	2.30 × 10^−14^
cg14598846	145008909	1.11 × 10^−22^	2.72 × 10^−19^
cg23299254	145008957	4.87 × 10^−23^	2.38 × 10^−19^
cg10299941	145009137	0.0001	0.04
14 [rs4764133/rs12316046] (12)	15114233	0.0002	0.04
cg20917083			
18 [rs6516886/rs2832155] (21)			
cg00065302	30366250	0.0001	0.04
cg05468028	30391383	1.53 × 10^−5^	0.006
cg18001427	30391784	2.95 × 10^−5^	0.01
cg20220242	30392188	3.92 × 10^−9^	2.40 × 10^−6^
cg24751378	30396349	3.27 × 10^−7^	0.0001
cg16140273	30455616	3.07 × 10^−5^	0.01

a *P* values are from all 87 samples combined. SNP = single‐nucleotide polymorphism; Chr. = chromosome; FDR = false discovery rate.

Many OA genetic association signals have only been discovered following stratification by joint and/or sex 5, 6. We repeated the mQTL analysis of all 14 loci using such stratification, but did not identify any further genotype–methylation correlations. Furthermore, to assess whether the identified mQTLs were more specific to OA than femoral neck fracture, we repeated the mQTL analysis by removing the 16 samples from the patients with femoral neck fracture. This analysis did not identify any OA‐specific or femoral neck fracture–specific genotype–methylation correlations. In summary, we identified 4 OA susceptibility loci demonstrating significant mQTLs, and these are discussed in more detail below.

#### Locus 7

Genotype at rs10471753 correlated with methylation of 1 CpG site, cg25008444, which is located 233 kb from the association SNP. This CpG site is within the gene body of *PIK3R1*, coding for phosphatidylinositol 3‐kinase regulatory subunit ɑ (Supplementary Figure 1A, available on the *Arthritis & Rheumatology* web site at http://onlinelibrary.wiley.com/doi/10.1002/art.40849/abstract). The OA risk–conferring C allele of rs10471753 correlated with higher methylation of cg25008444 (Supplementary Figure 1B).

#### Locus 11

**Figure 1:**
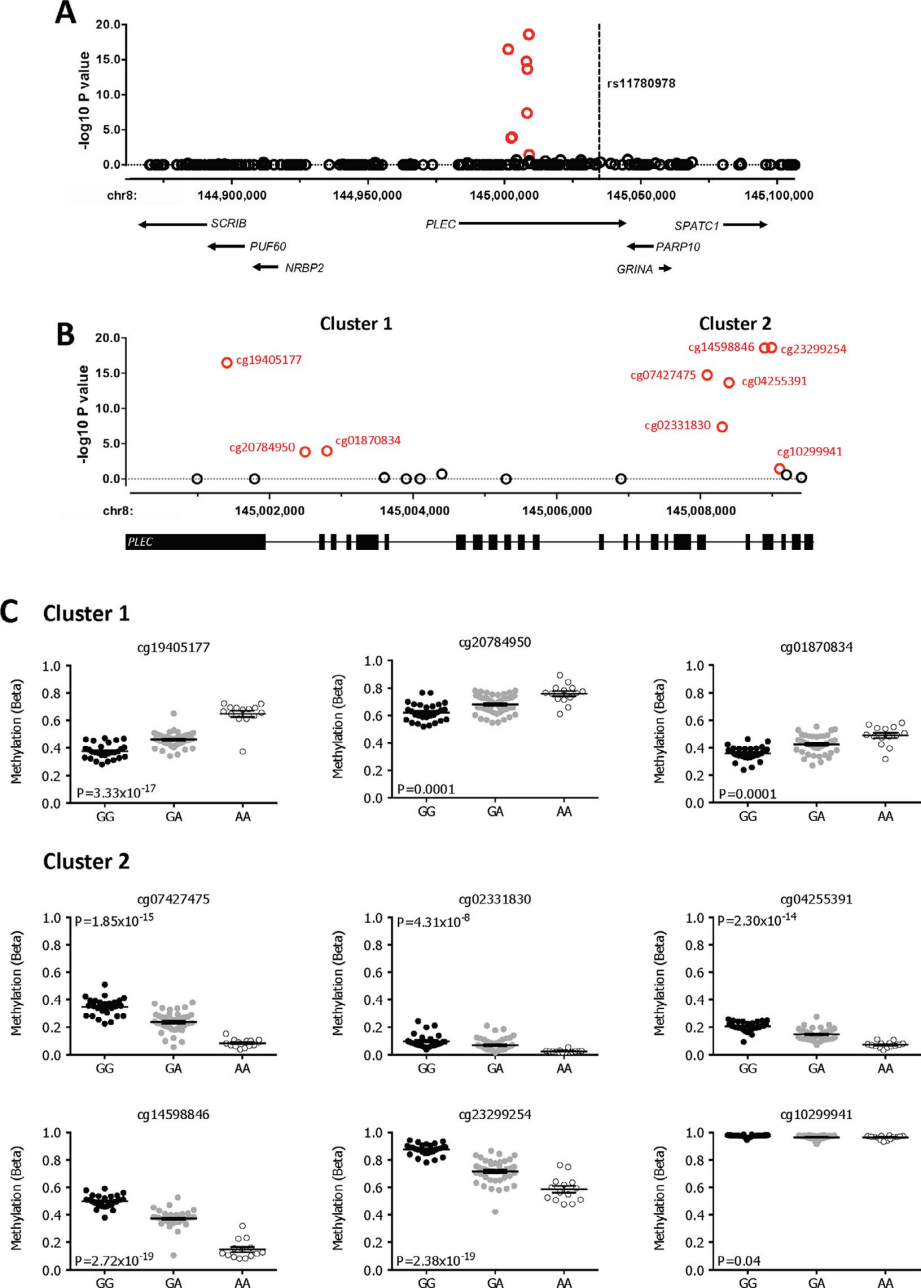
Correlation of genotype at rs11780978 with the methylation status of 9 CpG sites within the gene body of *PLEC*. **A**, The association between rs11780978 and methylation levels of CpG probes that are present within the region. The x‐axis represents the genomic position of the CpG probes, and the y‐axis represents the Benjamini‐Hochberg–corrected −log_10_ *P* value of the correlation between rs11780978 genotype and M value at each CpG probe. Each circle represents a single CpG probe, with the 9 significant associations highlighted in red. The broken line indicates the location of rs11780978. The genes within the region analyzed are indicated below the association plot, with the gene direction indicated by **arrows**. **B**, An enlarged image of *PLEC*, highlighting the location of the 9 significant CpG associations (red) falling into 2 distinct clusters. Each circle represents a single CpG probe. **C**, The association between genotype at rs11780978 and methylation levels at the 9 significant CpG probes for all 87 samples. The level of methylation at the CpG probes is shown as the β value. Symbols represent individual patient samples. Horizontal lines and error bars show the mean ± SEM.

Genotype at rs11780978 correlated with methylation of 9 CpG sites located within an interval of only 7.7 kb and positioned between 25.7 kb and 33.4 kb from the association SNP. The 9 CpG sites are all within the gene body of *PLEC*, which encodes plectin (Figure 1A). They form 2 clusters as follows: cg19405177, cg20784950, and cg01870834 are located within an interval of 1.4 kb, followed by a gap of 5.3 kb (which contains 6 CpG sites covered by the array and not demonstrating an mQTL) and then 6 CpG sites (cg07427475, cg02331830, cg04255391, cg14598846, cg23299254, and cg10299941), which are located within an interval of 1.0 kb (Figure 1B). For each of the 3 CpG sites within the first cluster, the OA risk–conferring A allele of rs11780978 correlated with higher methylation, while for each of the 6 CpG sites within the second cluster, the A allele correlated with lower methylation (Figure 1C).

#### Locus 14

Genotype at rs4764133 correlated with methylation at 1 CpG site, cg20917083, which is located 50 kb from the association SNP. This CpG is within an intron of *ARHGDIB*, which codes for Rho GDP dissociation inhibitor 2 (Supplementary Figure 2A, available on the *Arthritis & Rheumatology* web site at http://onlinelibrary.wiley.com/doi/10.1002/art.40849/abstract). The OA risk–conferring T allele of rs4764133 correlated with higher methylation of cg20917083 (Supplementary Figure 2B).

#### Locus 18

Genotype at rs6516886 correlated with methylation at 6 CpG sites. These are located at either side of the SNP, with cg00065302 (27.4 kb from the SNP), cg05468028 (2.3 kb from the SNP), cg18001427 (1.9 kb from the SNP), and cg20220242 (1.5 kb from the SNP) located on the centromeric side of rs6516886, while cg24751378 (2.7 kb from the SNP) and cg16140273 (62 kb from the SNP) are located on its telomeric side (Supplementary Figure 3A, available on the *Arthritis & Rheumatology* web site at http://onlinelibrary.wiley.com/doi/10.1002/art.40849/abstract). The CpG site cg00065302 is intergenic and located between *LTN1*, which codes for E3 ubiquitin‐protein ligase listerin, and *RWDD2B*, which codes for RWD domain–containing protein 2B. The CpG site cg05468028 is located in the gene body of *RWDD2*, while cg18001427 and cg20220242 are located immediately upstream of this gene. The CpG site cg24751378 is intergenic and located between and upstream of *RWDD2B* and *USP16*, which codes for ubiquitin carboxyl‐terminal hydrolase 16. The CpG site cg16140273 resides within an intron of *MAP3K7CL*, which codes for MAP3K7 C‐terminal–like protein. The OA risk–conferring T allele of rs6516886 correlated with increased methylation of cg00065302, cg05468028, cg18001427, cg20220242, and cg24751378, and with decreased methylation of cg16140273 (Supplementary Figure 3B).

#### Focus on locus 11

Based on the large number of highly significant CpG sites from within locus 11, which all cluster in an interval of only 7.7 kb, we focused our attention on rs11780978 and set out to further investigate this association signal. As noted above, these CpG sites reside within *PLEC* at chromosome 8q24.3. This region of the genome is gene rich. By analyzing the GTEx database, we first assessed whether there were any known rs11780978 eQTLs operating on genes within the locus. This was the case in a broad range of cell types for *PLEC* and also for *PARP10*,* GRINA,* and *SPATC1*, which are located in the immediately telomeric region of *PLEC* (Figure 1B). The GTEx database does not include data on cartilage tissue samples. Using our RNA‐Seq data, we observed expression of *PLEC*,* PARP10,* and *GRINA* in cartilage (TPMs >10) (Supplementary Figure 4A, available on the *Arthritis & Rheumatology* web site at http://onlinelibrary.wiley.com/doi/10.1002/art.40849/abstract), but not of *SPATC1* (TPM <1). Each of these 3 cartilage‐expressed genes showed significant differential expression between our OA and femoral neck fracture cartilage samples, with *PLEC* and *PARP10* demonstrating increased expression in OA (*P* = 0.014 and *P* = 0.002, respectively) and *GRINA* demonstrating reduced expression (*P* = 0.028). Ensembl (http://www.ensembl.org/index.html) lists 15 transcript isoforms for *PLEC*, 23 for *PARP10*, and 10 for *GRINA*. The majority of these were detectable in our RNA‐Seq data for both the OA and femoral neck fracture cartilage samples (Supplementary Figures 4B–D).

#### Methylation QTL, eQTL, and methylation‐expression QTL analysis in new patients

**Figure 2:**
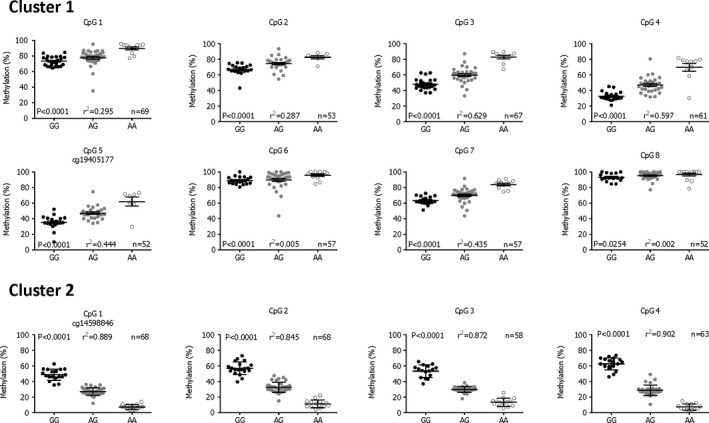
Association between rs11780978 genotype and methylation for *PLEC* CpG sites cg19405177 and cg14598846 in the new osteoarthritis patients (n = 104). In cluster 1, the pyrosequencing assay targeting cg19405177 (CpG5) captured 7 additional CpG sites (CpG1–CpG4 and CpG6–CpG8) located between 67 bp upstream and 45 bp downstream of cg19405177. In cluster 2, the pyrosequencing assay targeting cg14598846 (CpG1) captured 3 additional CpG sites (CpG2–CpG4) located up to 22 bp downstream of cg14598846. *P* values were calculated using the Kruskal‐Wallis test. The square of the correlation coefficient (r^2^) values were calculated using a model of standard least squares. Horizontal lines and error bars show the mean ± SEM. n = the number of patients providing data per CpG.

We assessed whether cartilage expression of *PLEC*,* PARP10,* or *GRINA* correlated with rs11780978 genotype and, if so, whether any such eQTLs correlated with methylation. As a first step, we recruited additional OA patients undergoing arthroplasty and extracted DNA and RNA samples from their cartilage. Using pyrosequencing, we measured methylation levels at the *PLEC* CpG sites in the DNA samples. We focused on 2 of the most significant CpG sites: cg19405177 from the cluster of 3 (cluster 1) and cg14598846 from the cluster of 6 (cluster 2) (Table 2 and Figure 1). Each pyrosequencing assay captured the relevant CpG site plus additional CpG sites located nearby: 7 sites for cg19405177 and 3 sites for cg14598846. Stratification by rs11780978 genotype replicated our initial mQTL result, and the OA risk–conferring A allele of rs11780978 correlated with higher methylation at cg19405177 (and its 7 flanking CpG sites) and with lower methylation at cg14598846 (and its 3 downstream CpG sites) (Figure 2).

**Figure 3:**
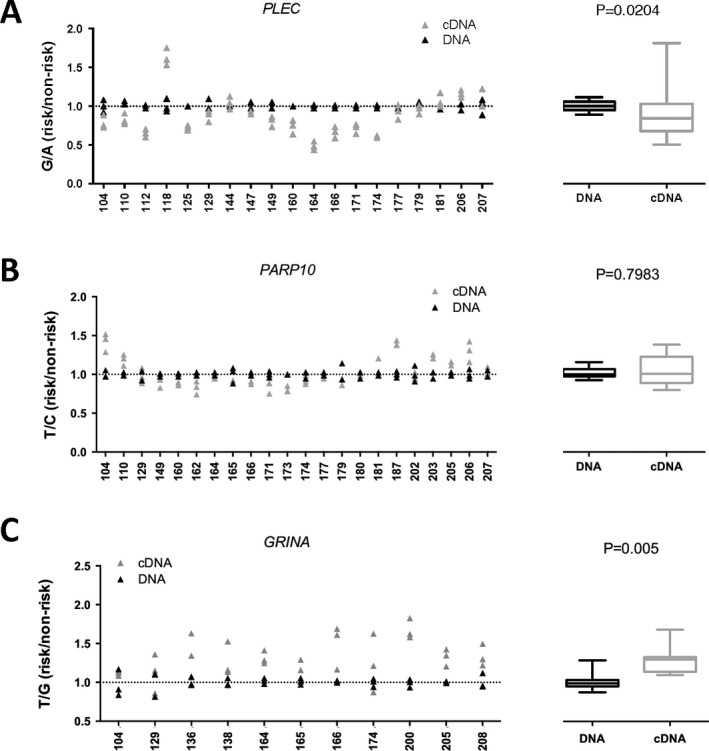
Allelic expression imbalance (AEI) analysis in cartilage from new osteoarthritis patients (n = 104). AEI analysis was conducted for *PLEC* using transcript single‐nucleotide polymorphism (SNP) rs11783799 (**A**), for *PARP10* using transcript SNP rs11136345 (**B**), and for *GRINA* using transcript SNP rs9100 (**C**). The risk/nonrisk allelic ratio is plotted, with a ratio of <1 indicating decreased expression of the risk allele. Three technical repeats were performed for each patient's DNA sample (black) and cDNA sample (gray). Numbers on the x‐axis refer to the anonymized identification number assigned to patients at recruitment. The mean values for DNA and cDNA are combined (left panels), with results represented as box plots, in which the lines within the box represent the median, the box represents the 25th to 75th percentile, and the whiskers represent the maximum and minimum values (right). *P* values were calculated using Wilcoxon's matched pairs signed rank test.

We next tested for eQTLs by undertaking AEI analysis in the new patients. For each gene, we selected a transcript SNP that was in linkage disequilibrium with rs11780978, and we identified those rs11780978 heterozygotes that were also heterozygous for 1 or more of the transcript SNPs. AEI was then performed using cDNA synthesized from the cartilage RNA samples. For *PLEC* we used the transcript SNP rs11783799 (r^2^ = 0.93) and analyzed 19 compound heterozygotes. For *PARP10* we used the transcript SNP rs11136345 (r^2^ = 0.85) and analyzed 22 compound heterozygotes. For *GRINA* we used the transcript SNP rs9100 (r^2^ = 0.78) and analyzed 11 compound heterozygotes. Significant AEI was observed at *PLEC* and *GRINA*, but not at *PARP10* (Figure 3). For *PLEC*, the G allele of rs11783799, which correlates with the OA risk–conferring A allele of rs11780978, demonstrated reduced expression in all 19 compound heterozygotes combined (G:A ratio <1.0; *P* = 0.02). For *GRINA*, the T allele of rs9100, which correlates with the A allele of rs11780978, demonstrated increased expression in all 11 compound heterozygotes combined (T:G ratio >1.0; *P* = 0.005).

**Figure 4:**
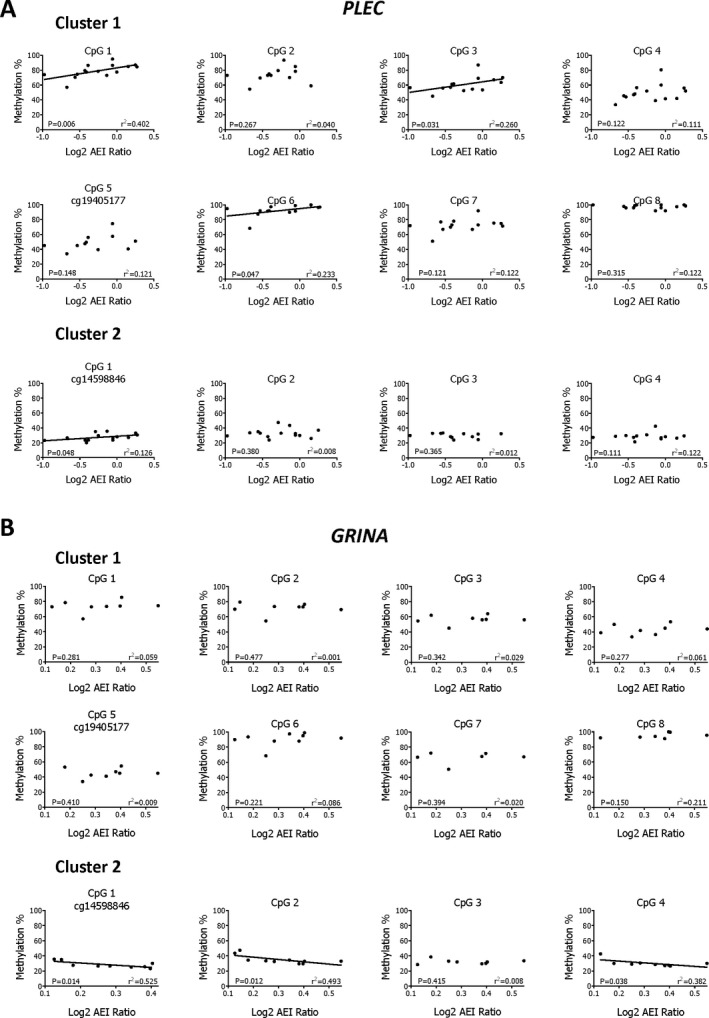
Methylation expression quantitative trait locus analysis of *PLEC* (**A**) and *GRINA* (**B**). **A**,*PLEC* log_2_ allelic expression imbalance (AEI) ratios and **B**,*GRINA* log_2_ AEI ratios were plotted against methylation at cg19405177 and its 7 additional CpG sites (cluster 1) and at cg14598846 and its 3 additional CpG sites (cluster 2).

We plotted the *PLEC* and *GRINA* AEI data for the compound heterozygotes against their individual methylation values. We focused on the *PLEC* heterozygotes for which we had matched methylation data (up to 15 of the 19 heterozygotes). Additionally, we focused on the *GRINA* heterozygotes for which we also had methylation data (up to 10 of the 11). For *PLEC*, 3 cluster‐1 CpG sites demonstrated significant correlation between methylation and AEI: CpG1 (*P* = 0.006), CpG3 (*P* = 0.031), and CpG6 (*P =* 0.047) (Figure 4A). Additionally, we identified a significant correlation between methylation and *PLEC* AEI at a single cluster‐2 CpG site (CpG1; *P =* 0.048). For *GRINA*, there were no correlations with cluster‐1 CpG sites, but 3 cluster‐2 CpG sites demonstrated significant correlations: cg14598846 (*P* = 0.014), CpG2 (*P* = 0.012), and CpG4 (*P* = 0.038) (Figure 4B).

In summary, this analysis replicated the mQTLs, identified cartilage eQTLs at *PLEC* and *GRINA*, and revealed the existence of cartilage methylation‐expression QTLs (meQTLs) operating on both of these genes but correlating with distinct CpG sites. Methylation at both cluster 1 and 2 was associated with *PLEC* expression, while only methylation at cluster 2 correlated with *GRINA* expression.

#### A genetic difference between clusters 1 and 2

We next sought to assess whether there was a genetic basis for this difference in activity between cluster 1 and cluster 2 CpG sites. Using our original 87 patients, we determined whether any SNPs on the array that were part of the same linkage disequilibrium block as the association signal rs11780978 showed a more significant genotype–methylation correlation with cg19405177 or cg14598846. For cg19405177, we identified rs7819099 (r^2^ = 0.83, uncorrected *P* = 1.66 × 10^−28^ versus 2.04 × 10^−20^ for rs11780978), and for cg14598846 we identified rs11136336 (r^2^ = 0.94, uncorrected *P* = 3.69 × 10^−27^ versus 1.11 × 10^−22^ for rs11780978). Both SNPs are also located within *PLEC* and have a pairwise r^2^ value of 0.87. We genotyped these 2 SNPs in our new patients (n = 104) and, as in the 87 patients, we correlated genotype with methylation. In Supplementary Figure 5 (available on the *Arthritis & Rheumatology* web site at http://onlinelibrary.wiley.com/doi/10.1002/art.40849/abstract), the data are presented as a heatmap for the 87 array patients and for the 104 new patients. For cg19405177, rs7819099 also showed a stronger effect than rs11780978 in the new patients, and in the 2 patient groups combined. For cg14598846, rs11136336 and rs11780978 were more comparable in the new patients and in the 2 groups combined. Overall, these data reveal different polymorphisms as drivers of the methylation–genotype correlations observed at the 2 clusters: rs7819099 for cluster 1 and rs11136336/rs11780978 for cluster 2.

#### Interaction between the CpG clusters and nearby genes

Finally, we used in silico data and the WashU epigenome browser to search for chromatin interactions between the 2 clusters and nearby genes. No cartilage or chondrocyte data were available for the locus, but we identified relevant data for the human breast cancer cell line MCF7, the chronic myeloid leukemia cell line K562, and the human lymphoblastoid cell line GM12878. For each of these cell lines there was evidence of interaction between the clusters and upstream sequences of several *PLEC* isoforms (Supplementary Figure 6, available on the *Arthritis & Rheumatology* web site at http://onlinelibrary.wiley.com/doi/10.1002/art.40849/abstract). These data indicate that, at least in these cell lines, these regions harboring the CpG sites physically interact with the *PLEC* gene.

## Discussion

Using genome‐wide data, we identified 4 additional OA susceptibility loci in which the risk‐conferring allele correlates with differential DNA methylation relative to the nonrisk allele *in cis*. Combined with our previous analyses 10, 12, we have now identified 8 such mQTL loci out of a total of 30 OA association signals studied, which is a frequency of >25%. We suspect that this is an underestimation, considering the low CpG coverage of the Illumina methylation array used (~1.5% of the total CpG sites in the human genome) and our relatively modest sample size of 87 patients. Our analysis, therefore, emphasizes the potentially pivotal mechanistic role that DNA methylation plays in the activity of many OA genetic risk loci, and now warrants targeted editing of the epigenome in order to determine causality. Furthermore, it highlights that this effect is operating on cartilage, the tissue central to the disease process, and is detectable at a clinically relevant point for the patient, namely, when arthroplasty is required.

Of the 4 novel mQTL loci that we report here as significant following FDR correction, we chose to focus on locus 11 because of the large number of highly significant CpG sites at this signal, i.e., 5 of the 9 positive CpG sites had FDR *P* values of <1 × 10^−10^. A subsequent analysis revealed eQTLs operating on genes within the locus. There is evidence of eQTLs correlating with the OA association SNPs at the other 3 loci (data not shown), and these signals, therefore, also merit follow‐up studies.

Methylation QTLs operating at the locus 11 CpG sites in association with the OA SNP, or other variants in high linkage disequilibrium with it, have been previously reported in a range of tissues including adipose, pancreas, lung, lymphocytes, and fibroblasts 34, 35, 36, 37, 38, 39. However, this is the first study of these mQTLs operating in cartilage, and the first study to identify a link between these CpG sites and OA genetic risk. Hypothesizing that the mQTL at this locus signified an eQTL of functional relevance to the association signal, we used a combination of in silico and in vitro analyses to highlight *PLEC* and *GRINA* as likely target genes of the rs11780978 association signal.

*PLEC* codes for plectin, a multifunctional cytoskeletal linker protein that directly interacts with a broad range of cytoplasmic, membrane, and organelle proteins 40, 41. Plectin has a range of functions, including acting as a scaffold for signaling proteins and as an organizer of cytoskeletal filaments. The protein is particularly abundant in tissues that are subjected to mechanical stress, and most research to date focuses on plectin in skin, muscle, and blood vessels. Mutations of *PLEC* result in skin blistering and muscular dystrophy, with the tissue affected and the severity of the disease being determined by the site of the mutation 41. *GRINA* is a member of a family of genes coding for 6 proteins named “transmembrane BAX inhibitor motif–containing,” (TMBIM). These genes encode calcium channels that are present in the Golgi complex, ER, and mitochondria, where they control calcium homeostasis 42. By controlling calcium flow, the TMBIMs regulate cell death, including during ER stress, with the majority of the proteins being antiapoptotic 43. *GRINA* encodes TMBIM3, which is expressed in most cells, and localizes to the Golgi complex and ER, where it suppresses ER stress–induced apoptosis. To the best of our knowledge, plectin and TMBIM3 have not been functionally linked to an arthritis phenotype before.

Our AEI data indicate that the OA risk–conferring A allele of rs11780978 correlates with reduced expression of *PLEC* but with increased expression of *GRINA*, while our RNA‐Seq data revealed increased *PLEC* expression but decreased *GRINA* expression, in OA versus non‐OA (femoral neck fracture) cartilage. For *PLEC*, our interpretation of this is that in an OA joint, additional plectin is required to mitigate the effect of an altered biomechanical load. However, inheritance of the A allele, with its concomitant lower expression of *PLEC*, attenuates this mitigation. For *GRINA*, our interpretation is that an alteration in the activity of cells in the OA joint necessitates a reduction in *GRINA* expression to facilitate controlled cell death. However, the A allele, with its concomitant increased *GRINA* expression, hinders this response.

One of the most striking outcomes of our investigation was the discovery of 2 clusters of CpG sites that were physically close but had quite separate and distinct characteristics as follows: 1) for cluster 1 the risk‐conferring A allele of rs11780978 correlated with higher methylation, whereas for cluster 2 it correlated with lower methylation; 2) CpG sites in both cluster 1 and cluster 2 correlated with an meQTL operating on *PLEC*, whereas only cluster‐2 CpG sites correlated with an meQTL operating on *GRINA*; and 3) methylation at cluster‐1 CpG sites correlated more strongly with genotype at rs7819099, whereas methylation at cluster‐2 CpG sites correlated more strongly with genotype at rs11136336/rs11780978. Combined, these data support the notion that at least 2 functional effects are encoded by polymorphisms at the *PLEC* locus, mediated by methylation on distinct CpG sites and impacting 2 separate genes.

In conclusion, our analysis has identified novel mQTLs and highlighted the interplay between OA genetic risk, DNA methylation, and gene expression in cartilage. We have discovered relevant functional effects on 2 genes from within a single locus and, as such, have elevated these to prime targets for the encoded risk at this locus. These genes and their encoded proteins now merit much more detailed investigation in the context of OA etiology.

## Author contributions

All authors were involved in drafting the article or revising it critically for important intellectual content, and all authors approved the final version to be submitted for publication. Drs. Rice and Loughlin had full access to all of the data in the study and take responsibility for the integrity of the data and the accuracy of the data analysis.

### Study conception and design

Rice, Shepherd, Reynard, Loughlin.

### Acquisition of data

Rice, Tselepi, Sorial, Aubourg, Shepherd, Skelton, Pangou, Deehan, Reynard, Loughlin.

### Analysis and interpretation of data

Rice, Tselepi, Sorial, Aubourg, Shepherd, Almarza, Skelton, Reynard, Loughlin.
