# Supplementary figures and images for: Prioritization of PLEC and GRINA as Osteoarthritis Risk Genes Through the Identification and Characterization of Novel Methylation Quantitative Trait Loci
Source: Arthritis Rheumatol. 2019 Jun 27;71(8):1285–96. doi: 10.1002/art.40849 (PMC6790675; doi:10.1002/art.40849)

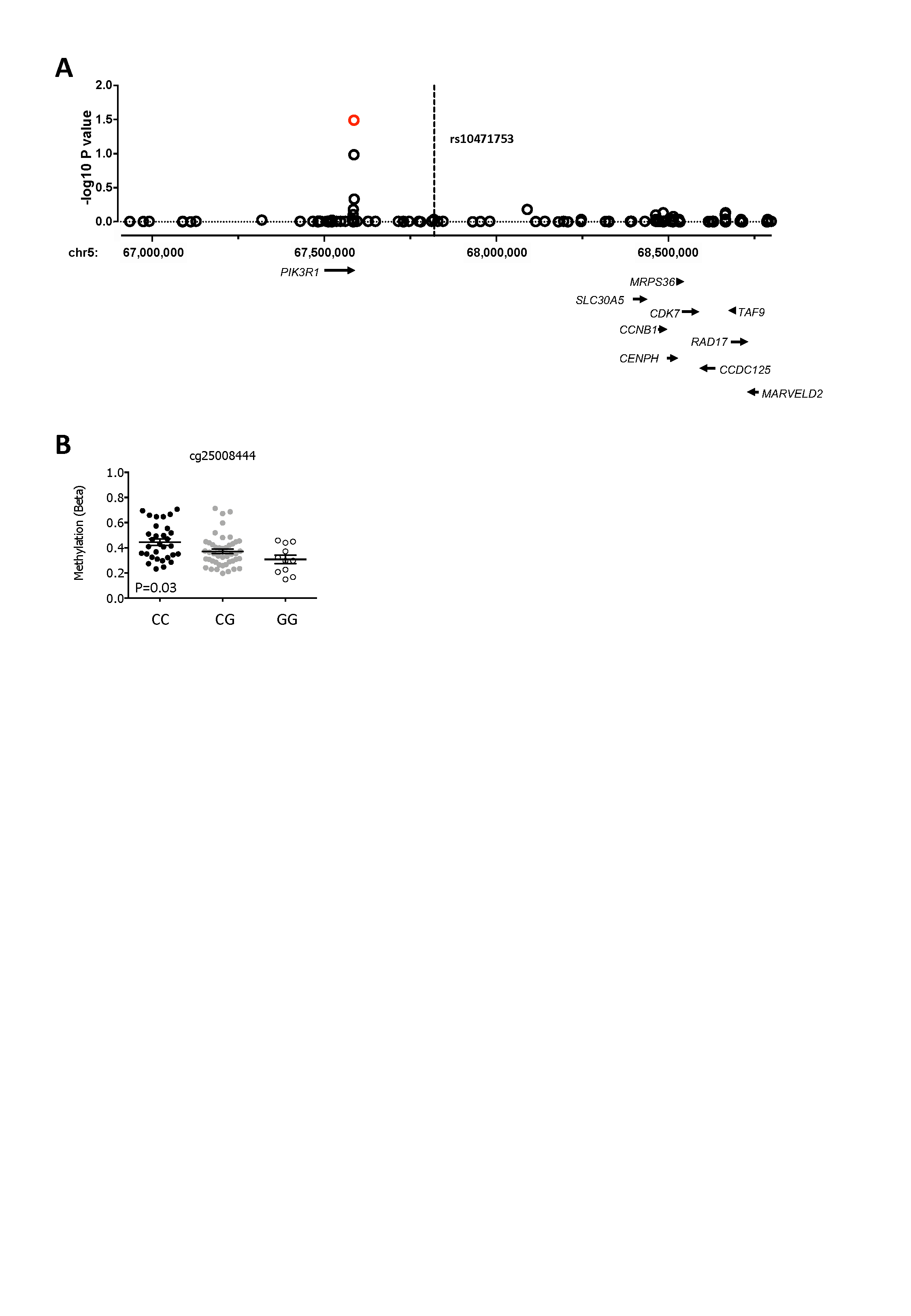

Supplement: Supplementary file 1 [file ART-71-1285-s001.tiff]

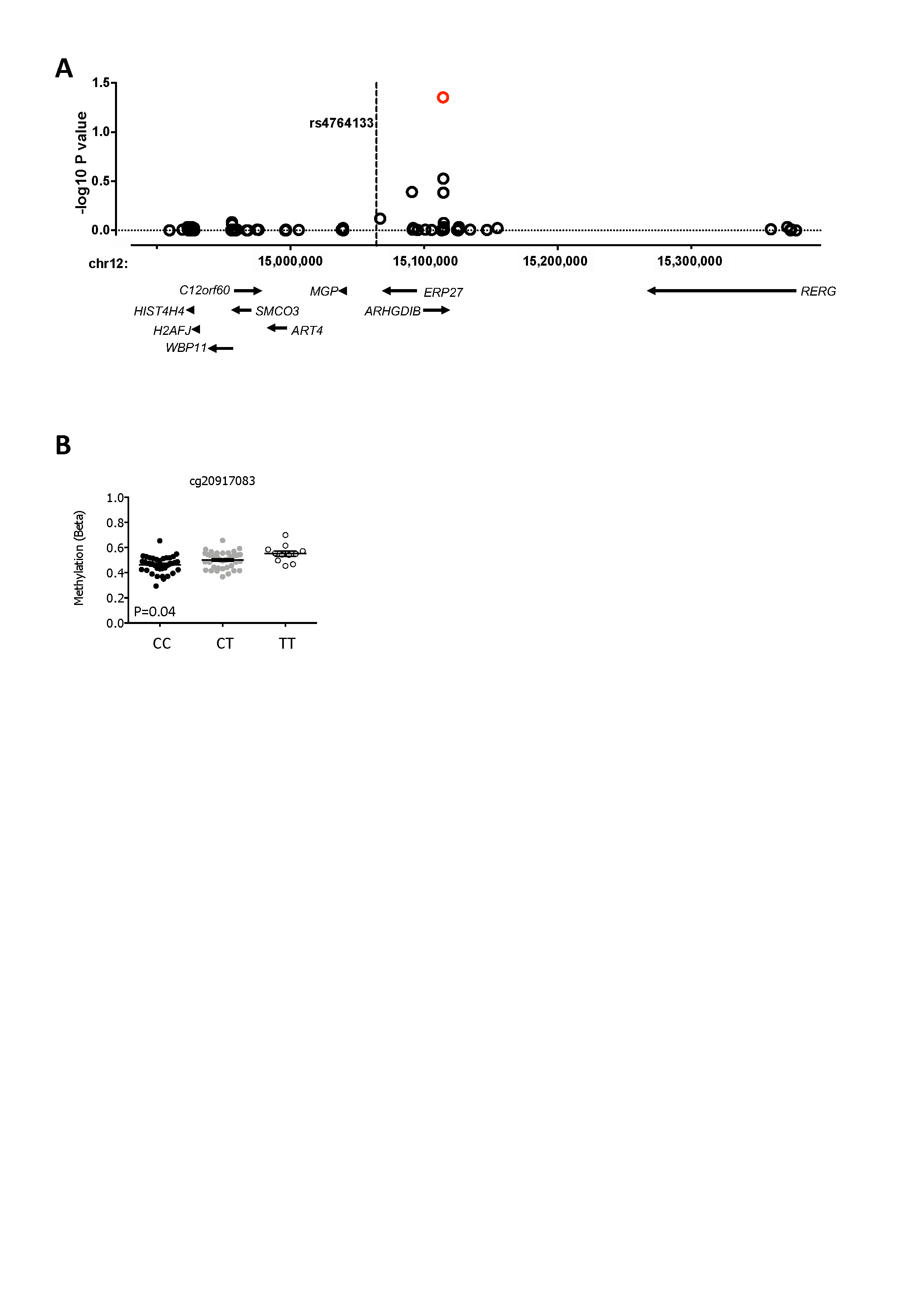

Supplement: Supplementary file 2 [file ART-71-1285-s002.tiff]

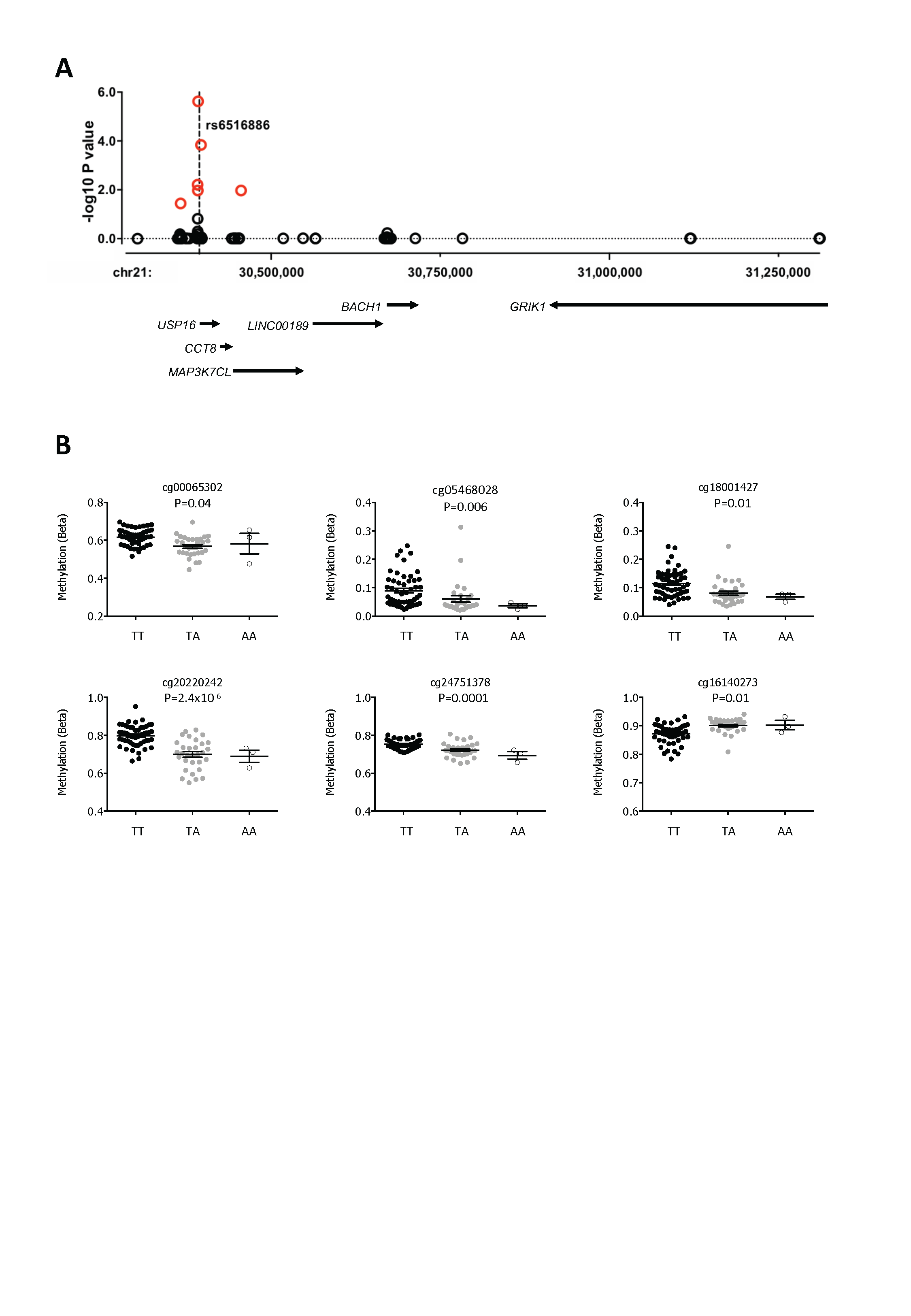

Supplement: Supplementary file 3 [file ART-71-1285-s003.tiff]

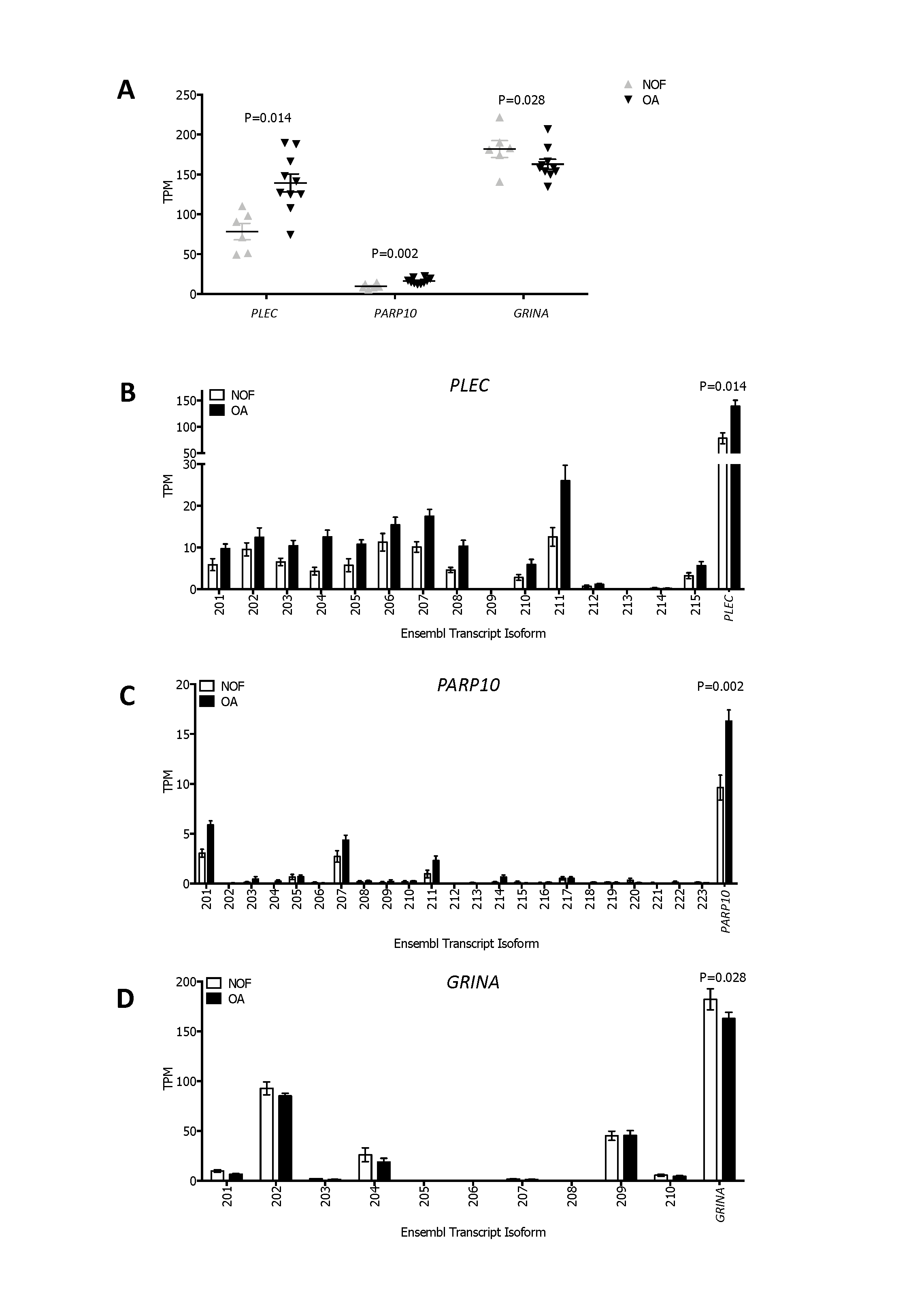

Supplement: Supplementary file 4 [file ART-71-1285-s004.tiff]

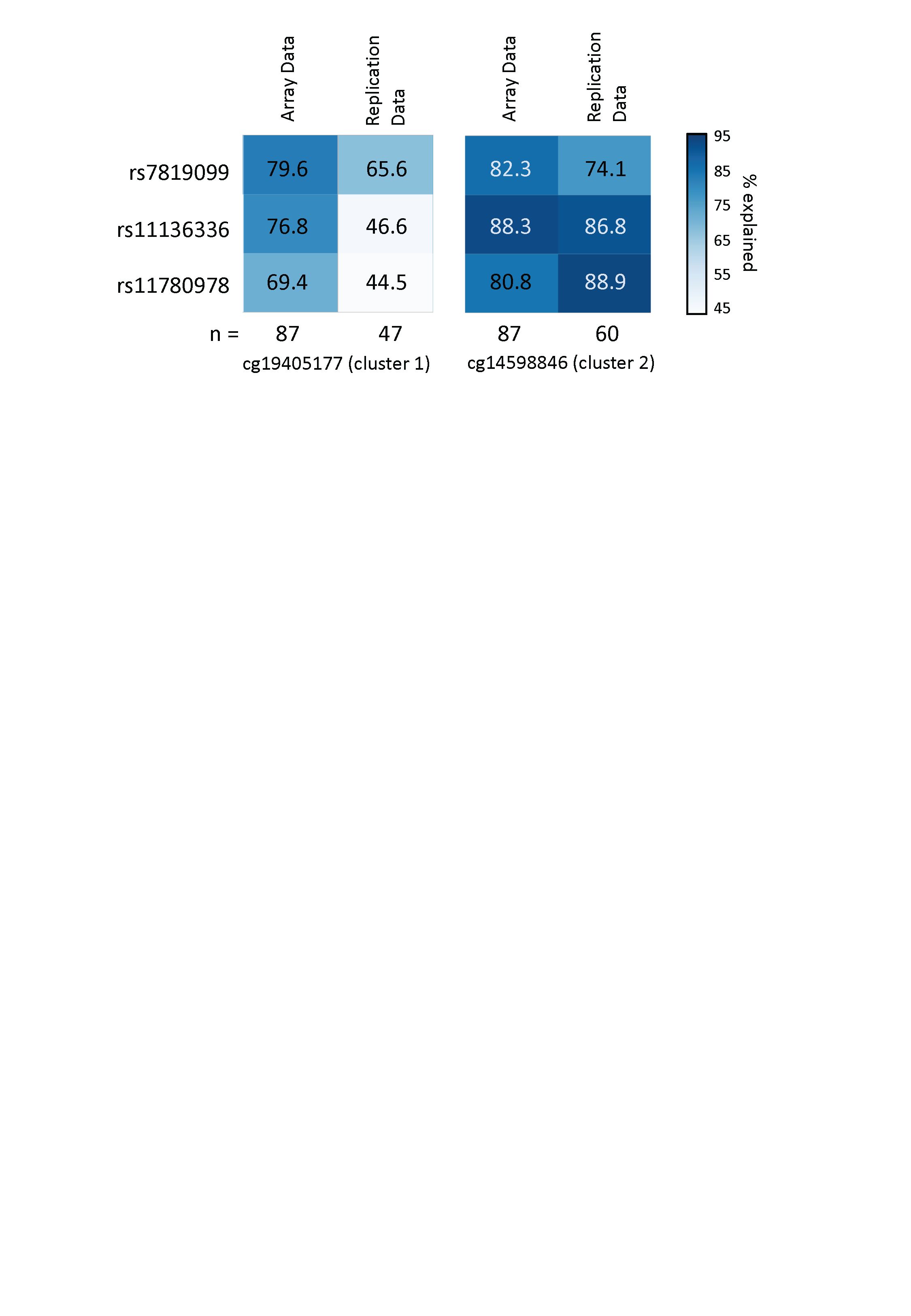

Supplement: Supplementary file 5 [file ART-71-1285-s005.tiff]

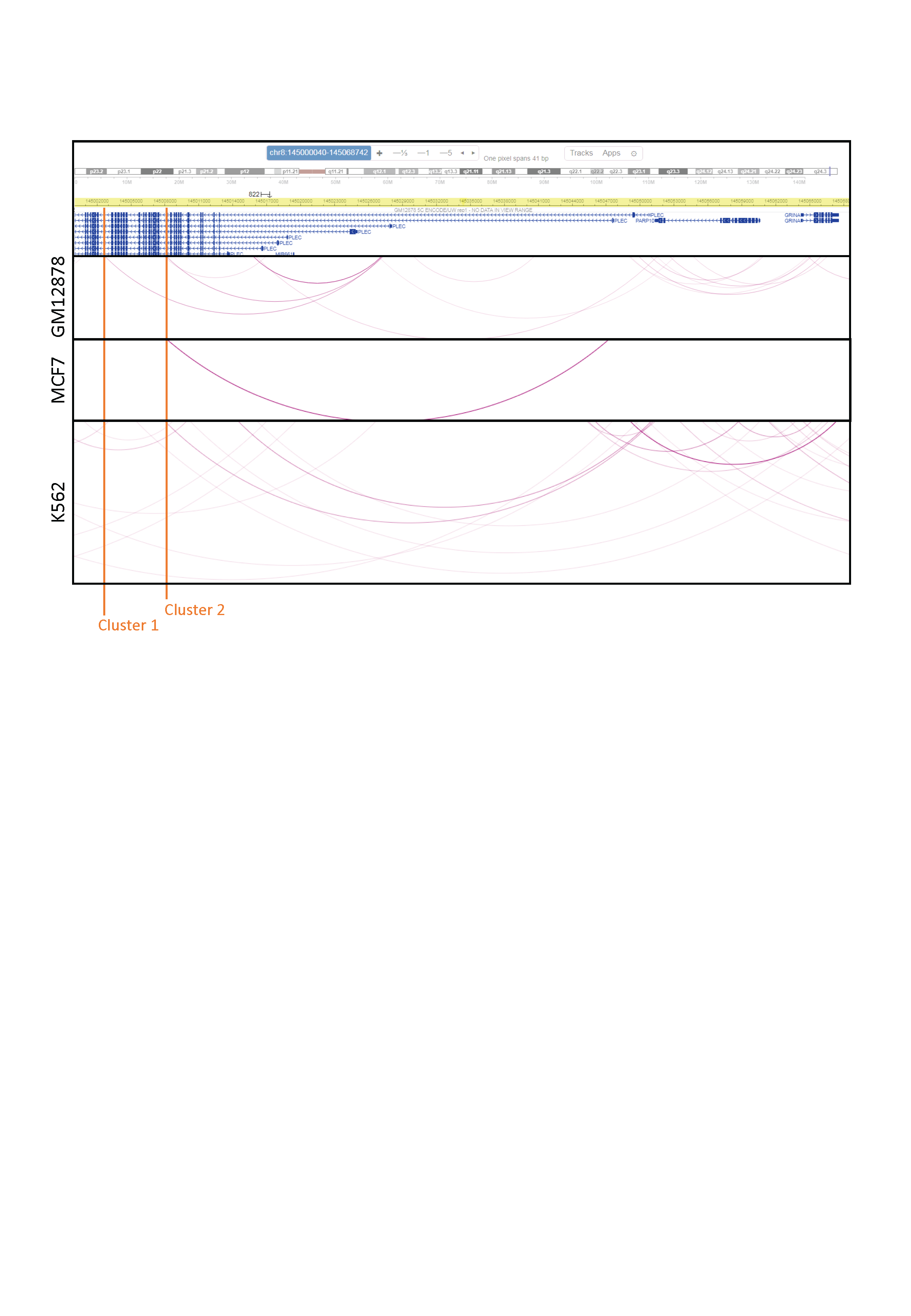

Supplement: Supplementary file 6 [file ART-71-1285-s006.tiff]
