# Supplementary material for: Prioritization of PLEC and GRINA as Osteoarthritis Risk Genes Through the Identification and Characterization of Novel Methylation Quantitative Trait Loci
Source: Arthritis Rheumatol. 2019 Jun 27;71(8):1285–96. doi: 10.1002/art.40849 (PMC6790675; doi:10.1002/art.40849)
Supplement: Supplementary file 8 [file ART-71-1285-s008.docx]

**Supplementary Table 2.** Primers used for genotyping SNPs. The rs11783799 and rs11136345 assays were also used for allelic expression imbalance (AEI) analysis at *PLEC* and *PARP10*, respectively. [Btn], biotin tag at the 5' end of the primer. n/a, not applicable.

| **SNP** | **Gene** | **Genotyping method** | **Forward primer (5'-3')** | **Reverse primer (5'-3')** | **Sequencing primer (5'-3')** | **Discriminatory enzyme** |
| --- | --- | --- | --- | --- | --- | --- |
| rs11780978 | *PLEC* | RFLP | TGGGTGATAAAGCAAGACTCTGT | GTGGGGTGGGTGGGAAAG | n/a | *NlaIII* |
| rs11783799 | *PLEC* | Pyrosequencing | [Btn]CCCACCCACCTCTCAAAC | GTCTACAGGGAGCGGTGCA | TGGCTACCACCCGCT | n/a |
| rs11136345  rs7819099 | *PARP10*  *PLEC* | Pyrosequencing  RFLP | [Btn]CTCAGCTGGGCACCATGT  ACAGCCTCATCCACAGTCAG | ACAGACGCCGAGAGGGTCTT  TCGAGACCTACAACCTGCTC | TTGGCCCAGGCAGAT  n/a | n/a  *HaeIII* |
