# Supplementary material for: Prioritization of PLEC and GRINA as Osteoarthritis Risk Genes Through the Identification and Characterization of Novel Methylation Quantitative Trait Loci
Source: Arthritis Rheumatol. 2019 Jun 27;71(8):1285–96. doi: 10.1002/art.40849 (PMC6790675; doi:10.1002/art.40849)
Supplement: Supplementary file 9 [file ART-71-1285-s009.docx]

**Supplementary Table 3.** Primers used for targeted methylation analysis by pyrosequencing. [Btn], biotin tag at the 5' end of the primer.

| **CpG** | **Forward primer (5'-3')** | **Reverse primer (5'-3')** | **Sequencing primer (5'-3')** |
| --- | --- | --- | --- |
| cg19405177 | ATGGTTTAGGTTTAGGGAAAGT | [Btn]CCATCACCCAAACCCTACTA | AGGTTTAGGGAAAGTT |
| cg14598846 | ATTTATTTTTAGGGATTGGTAGATGT | [Btn] AACCCAAATTCCCCTCCAACTTC | GGAATTGAGATTATAGGATTTGTTA |
