# Supplementary material for: Prioritization of PLEC and GRINA as Osteoarthritis Risk Genes Through the Identification and Characterization of Novel Methylation Quantitative Trait Loci
Source: Arthritis Rheumatol. 2019 Jun 27;71(8):1285–96. doi: 10.1002/art.40849 (PMC6790675; doi:10.1002/art.40849)
Supplement: Supplementary file 11 [file ART-71-1285-s011.docx]

**Supplementary Figure Legends**

**Supplementary Figure 1.** Genotype at rs10471753 (rs6893396*) correlates with the methylation status of a CpG within the gene body of *PIK3R1*. (**A**) The plot shows the association between rs10471753 and methylation levels of CpGs probes that are present within the region. The x-axis represents the genomic position of the CpG probes, and the y-axis represents the Benjamini-Hochberg corrected -log_10_ *P*-value of the correlation between rs10471753 genotype and M-value at each CpG probe. Each open circle represents a single CpG probe, and the significant association (cg25008444) is highlighted in red. The location of rs10471753 is indicated. The genes within the region analysed are indicated below the association plot, with the gene direction indicated by arrows. (**B**) The association between genotype at rs10471753 and methylation levels at cg25008444 for all 87 samples. The level of methylation at the CpG probe is shown as the β-value. Horizontal line represents the mean. *rs10471753 is not present on the HumanOmniExpress genotyping array and rs6893396, an array SNP that has an r^2^ of 1 with rs10471753, was therefore used to determine rs10471753 genotypes.

**Supplementary Figure 2.** Genotype at rs4764133 (rs12316046*) correlates with the methylation status of a CpG within the gene body of *ARHGDIB*. (**A**) The plot shows the association between rs4764133 and methylation levels of CpGs probes that are present within the region. The x-axis represents the genomic position of the CpG probes, and the y-axis represents the Benjamini-Hochberg corrected -log_10_ *P*-value of the correlation between rs4764133 genotype and M-value at each CpG probe. Each open circle represents a single CpG probe, and the significant association (cg20917083) is highlighted in red. The location of rs4764133 is indicated. The genes within the region analysed are indicated below the association plot, with the gene direction indicated by arrows. (**B**) The association between genotype at rs4764133 and methylation levels at cg20917083 for all 87 samples. The level of methylation at the CpG probe is shown as the β-value. Horizontal line represents the mean. *rs4764133 is not present on the HumanOmniExpress genotyping array and rs12316046, an array SNP that has an r^2^ of 0.98 with rs4764133, was therefore used to determine rs4764133 genotypes.

**Supplementary Figure 3.** Genotype at rs6516886 (*rs2832155) correlates with the methylation status of six CpGs within and intergenic to several genes. (**A**) The plot shows the association between rs6516886 and methylation levels of CpGs probes that are present within the region. The x-axis represents the genomic position of the CpG probes, and the y-axis represents the Benjamini-Hochberg corrected -log_10_ *P*-value of the correlation between rs6516886 genotype and M-value at each CpG probe. Each open circle represents a single CpG probe, and the six significant associations are highlighted in red. The location of rs6516886 is indicated. The genes within the region analysed are indicated below the association plot, with the gene direction indicated by arrows. (**B**) The association between genotype at rs6516886 and methylation levels at the six significant CpG probes for all 87 samples. The level of methylation at the CpG probes is shown as the β-value. Horizontal line represents the mean. *rs6516886 is not present on the HumanOmniExpress genotyping array and rs2832155, an array SNP that has an r^2^ of 1 with rs6516886, was therefore used to determine rs6516886 genotypes.

**Supplementary Figure 4.** Expression of *PLEC*, *PARP10* and *GRINA* in RNA-seq data from hip cartilage. (**A**) Comparison for each gene between the ten OA hip and six NOF samples. (**B-C**) Individual transcript isoform expression. ‘*PLEC’, ‘PARP10’* and *‘GRINA’* represent all isoforms combined, respectively. Bars represent the mean and the standard error of the mean (SEM) in (A), and the SEM in (B-C). *P*-values were calculated using a Wald test within the DESeq2 package.

**Supplementary Figure 5.** The effect of genotype upon methylation. Heat map displaying the percentage effect of genotype at rs7819099 (cluster 1 mQTL SNP), rs11136336 (cluster 2 mQTL SNP), and rs11780978 (association SNP) on methylation at cluster 1 CpG cg19405177 and cluster 2 CpG cg14598846. The relative contribution of each SNP upon methylation was determined using adjusted r^2^ values calculated using a standard least squares linear regression model and expressed as a percentage. The analysis was performed using SAS JMP Statistical Data Visualization Software. The data presented is for the 87 patients studied using the Illumina Infinium HumanMethylation450 array, and the new OA patients (n=47 for cg19405177 and 60 for cg14598846).

**Supplementary Figure 6.** A screenshot of the WashU Epigenome Browser displaying the identified long-range chromatin interactions associated with the two CpG clusters. The upper panel displays the genomic coordinates of the displayed region along with the physical location of the *PLEC, PARP10,* and *GRINA* transcripts falling within this region. ChIA-PET interactions were identified in GM12878, MCF7, and K563 cell lines and are displayed in the three lower panels. The location of the cluster 1 and cluster 2 CpGs is shown
